# Sinus-Plate Augmentation Method (SPAM) for Lateral Ridge Augmentation in the Maxillary Posterior Area: A Case Series

**DOI:** 10.3290/j.ohpd.c_2763

**Published:** 2026-08-26

**Authors:** Cosmin Dima, / Raluca Cosgarea

**Affiliations:** a Cosmin Dima Dentist and Surgeon, Dental Progress Center, Bucharest, Romania. Associate Researcher at University Victor Babes Timisoara, Romania. Performed all surgeries, wrote part of the manuscript.; b Raluca Cosgarea Professor, Department of Operative Dentistry and Periodontology, University of Bonn, Germany; Department of Periodontology and Peri-implant Diseases, University of Marburg, Germany; Associate Researcher at University Victor Babes Timisoara, Romania; Faculty of Dentistry, Iuliu-Hatieganu University, Cluj-Napoca, Romania. Analyzed the results, wrote the manuscript.

**Keywords:** autologous bone graft, dental implants, guided bone regeneration, maxillary augmentation, sinus lift.

## Abstract

**Purpose:**

This paper describes a novel surgical technique (Sinus-Plate Augmentation Method [SPAM]) using autologous sinus plate bone for posterior lateral maxillary augmentation during sinus lifting and dental implant insertion.

**Materials and Methods:**

Eight partially edentulous patients aged 52-71 years old (7 females, 2 smokers) with severe horizontal alveolar bone resorption in the maxillary posterior areas, were surgically treated as follows: lateral bone augmentation was performed by harvesting the vestibular bone wall of the sinus window and using a xenogeneic bone graft, followed by external sinus lifting and implant insertion. Seven patients were followed-up for 12-18 months, while one patient was followed-up for 36 months.

**Results:**

A total of 24 implants were inserted simultaneously with SPAM and sinus lifting. Post-operative healing was uneventful in all cases. Horizontal alveolar bone gain ranged between 9.28±1.2 mm post-operatively and 8.37±1.5 mm at the 19.5-month follow-up. A statistically significant gain in alveolar bone width of 2.55±1.47 mm was present at the follow-up.

**Conclusion:**

SPAM seems to be a feasible surgical technique for the horizontal reconstruction of moderate to severely resorbed posterior maxillae, concomitant to external sinus lifting and implant insertion.

Implant-prosthetic therapy plays a significant role in the reconstruction of edentulous areas. However, implant therapy in atrophic maxillary alveolar crests remains a challenge even for experienced oral surgeons and implantologists.^36^ Many patients demanding implant restorations in posterior maxillary areas have advanced vertical and lateral bone resorption and thus insufficient alveolar height for optimal implant positioning and close proximity to maxillary sinuses.^7^ The success of implant-prosthetic therapy for these patients depends on performing accurate dental implant planning, adequate surgical techniques for implant insertion and sinus lifting, and the optimal choice of grafting materials and augmentation procedures.^45^ In this context, patients edentulous in posterior maxillary areas often require sinus lifting combined with alveolar bone augmentation, to increase the bone volume needed for proper implant positioning.^5^

Lately, high survival rates for both short (93.91%) and longer dental implants placed simultaneously with alveolar bone augmentation (91.83%) have been reported for a minimum follow-up of 12 months. Nonetheless, less marginal bone loss (MBL) and more shallow probing pocked depths (PPD) were observed at short implants.^1^ Controversial reports on the success of short vs long implants combined with sinus lifting procedures without bone grafting are present: some authors report an uncertain long-term survival prognosis of 5-mm (short) implants in atrophic maxilla with a residual height between 4-6 mm. However, sinus lifting without bone grafting for posterior maxillary areas with 1-5 mm of residual bone height may suffice to regenerate new bone. Additionally, sinus lifting by the crestal approach in cases with residual alveolar bone of 3-6 mm and concomitant implant insertion (8 mm implants) may lead to fewer complications compared to the lateral window approach and 10-mm implants.^17,18^ However, a residual bone height of <5 mm seems to significantly decrease the implant survival rate.^3^ Other authors report comparable outcomes after 1 year for short implants in the atrophic posterior maxillary region and 10-mm-long implants in augmented alveolar bone (crestal approach).^19^

New technologies (e.g., piezoelectric devices, hydraulic sinus lifts, reamer burs) were proposed to replace conventional surgical techniques for sinus lifting, but these lack evidence of effectiveness and safety.^19^ Therefore, conventional surgical techniques are mostly used, and according to the clinician’s experience and skills, the most suitable surgical technique is chosen.^19^ Maxillary sinus augmentation is one of the most predictable pre-prosthetic surgical procedures, despite its possible complications (e.g., sinus membrane perforation, bleeding, graft infections, sinus infections, sinusitis). Nonetheless, these complications can be minimized or prevented through knowledge of maxillary sinus anatomy or implementation of measures to avoid them.^42^

Lack of appropriate horizontal alveolar bone (due to atrophy, sequelae of periodontal disease, traumas, congenital malformations) requires bone augmentation for optimal implant placement. This can be achieved through various surgical techniques implemented in horizontal and vertical bone augmentation: guided bone regeneration, bone-grafting techniques, alveolar bone expansion,^10^ the split-crest technique,^39^ the edentulous ridge expansion (ERE) technique,^38^ the Khoury Shell technique,^25^ the Sausage technique,^44^ augmentation with onlay-bone grafts,24 and classical guided bone regeneration (GBR). Some of these techniques provide advantages such as more predictable bone regeneration in large defect cases,^25^ or the use of resorbable membranes to avoid a second surgical intervention.^44^ Additionally, data with predictable long-term results have been reported for several techniques.^20,23^ However, certain disadvantages must be considered, such as the need for a second surgical site for bone block harvesting with increased morbidity, fracture during the separation of the alveolar ridge, bone resorption in cases with very thin residual bone, or high infection rates in cases where the non-resorbable PTFE membranes become exposed.^38,44^ Moreover, these techniques often involve the use of autogenous bone grafts, either alone or in combination with bone substitutes (i.e., allogeneic, xenogeneic, alloplastic grafting biomaterials) and different barrier membranes with special requirements (resorbable natural collagen; synthetic polymers; human, porcine, bovine pericardial membranes; human acellular freeze-dried dermal matrix; non-resorbable dense-polytetrafluoroethylene [d-PTFE], expanded-polytetrafluoroethylene [e-PTFE], titanium mesh, titanium-reinforced polytetrafluoroethylene).^13,16,40,47^ The use of allogeneic, xenogeneic, and alloplastic substitutes offers several advantages when compared to autologous bone, including less patient morbidity and the absence of a second surgical site (donor area).^41^ However, autogenous bone is still considered the gold standard due to its osteoconductive, osteoinductive, and osteogenic properties.^27^ The greatest disadvantage of autogenous bone grafts is the need for a second oral surgical site such as the mandibular ramus or chin, or an extraoral donor site (e.g., the iliac crest) with subsequent increased morbidity risk involving general anesthesia, hospitalization, prolonged recovery, and higher costs).^14^ For optimal augmentation results and successful osseointegration, evidence from preclinical and clinical studies indicate that not only are the augmentation technique and surgical manipulation important, but also the patient, especially in cases with vitamin D deficiency where supplementation especially in patients with systemic conditions may improve the outcomes.^32^

To the best of our knowledge, no technique for horizontal bone augmentation concomitant to sinus lifting and multiple implant placement using the external bony sinus wall has been described so far.

Therefore, the aim of this case series was to present a novel surgical technique (sinus-plate augmentation method, SPAM) for concomitant lateral/horizontal bone augmentation and sinus lifting for the implant insertion in the maxillary posterior area. The study hypothesis was that the lateral sinus bony wall can be successfully used for lateral augmentation and simultaneous sinus lifting and implant insertion in cases with moderate to severe lateral bone resorption.

## MATERIALS AND METHODS

### Patients

Seven female patients and one male patient aged 52-71 years old presenting moderate to severe horizontal bone resorption (mild to moderate Class C resorption^4^) in the edentulous maxillary right and/or left lateral area(s) with clinical indication for implant placement with simultaneous sinus lifting and lateral ridge augmentation, who were not heavy smokers (≥10 cigarettes/day) and were in good general health (i.e., exclusion criteria were radiation treatment in head and neck area, cardiovascular diseases requiring antibiotic prophylaxis, organ transplantation, infectious diseases) were included the current study.

All patients gave their informed written consent for performing the surgical procedures as well as documenting and publishing the results. No ethical approval was required for this case series.

### Surgical Procedure

All surgical procedures were performed under local anesthesia (Ultracain Forte, Normon Laboratories; Madrid, Spain) by a single experienced oral surgeon (CD) in a private dental practice (Figs 1a to 1c).

**Fig 1b:**
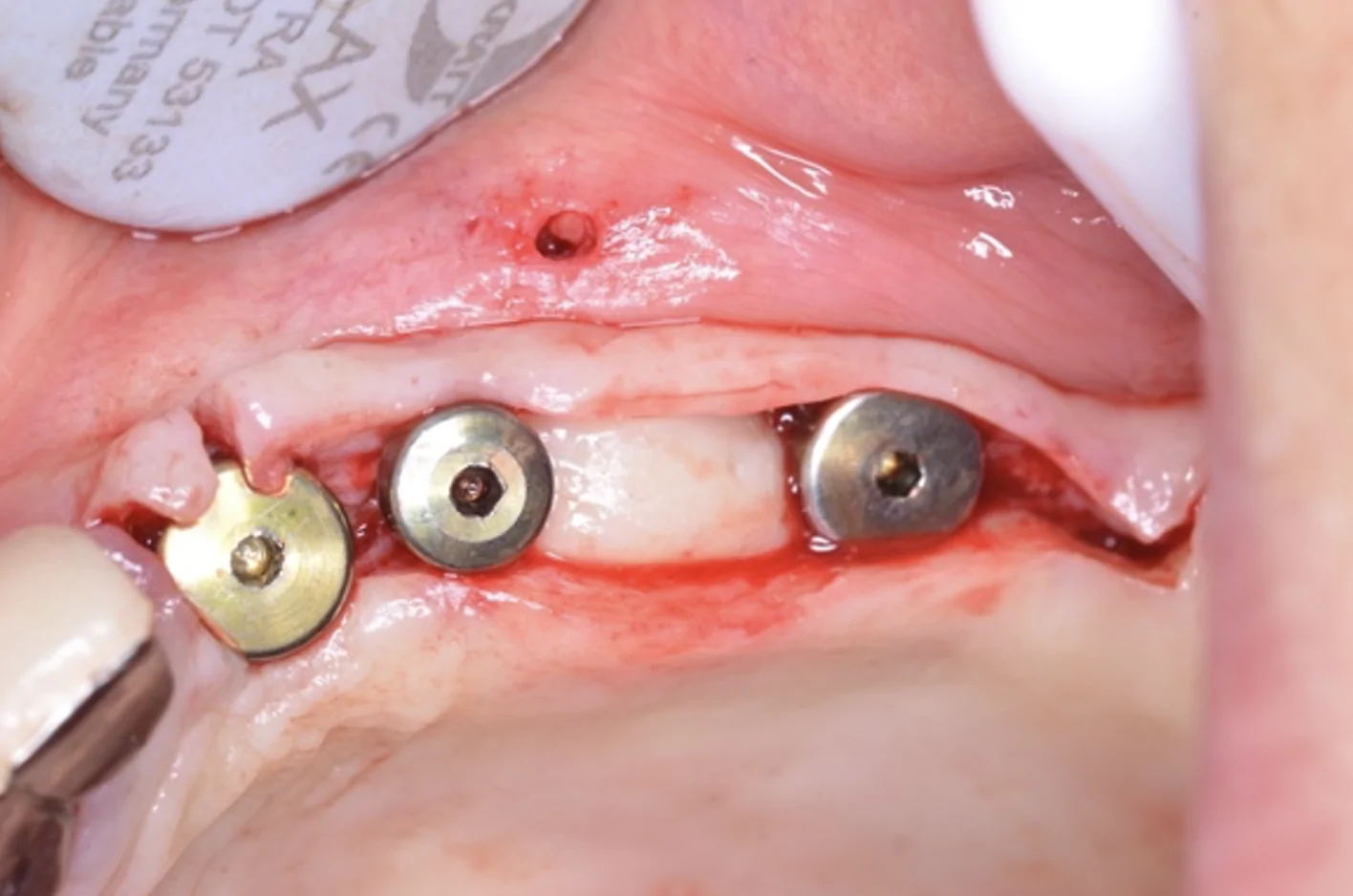
Intraoperative view at the second stage (implant uncovering).

**Fig 1c:**
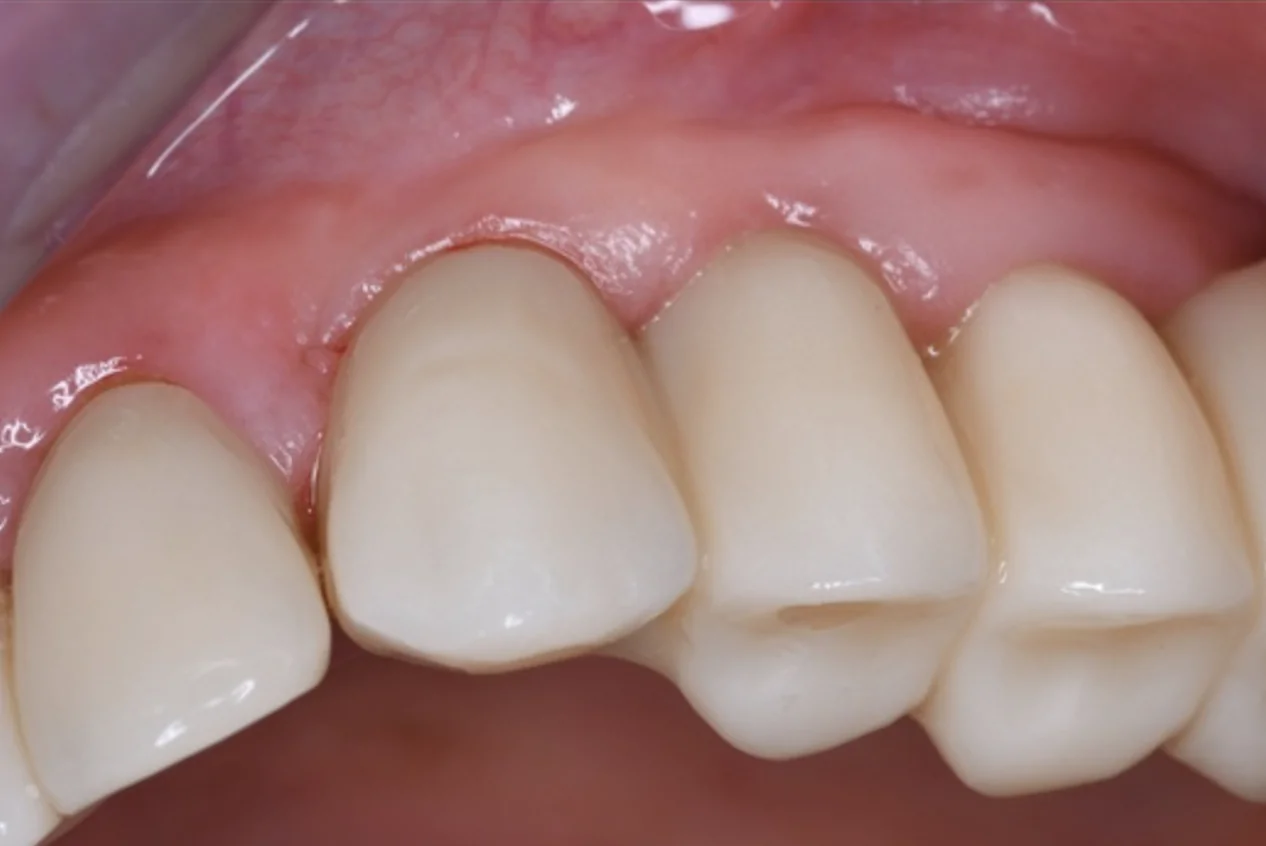
Clinical situation at follow-up (18 m post-operative).

Preoperatively, thorough clinical and radiographic (CBCT) evaluation as well as implant planning were performed using OnDemand3D software (CyberMed; Daejeon, Korea) (Figs 1 to 8).

**Fig 2b:**
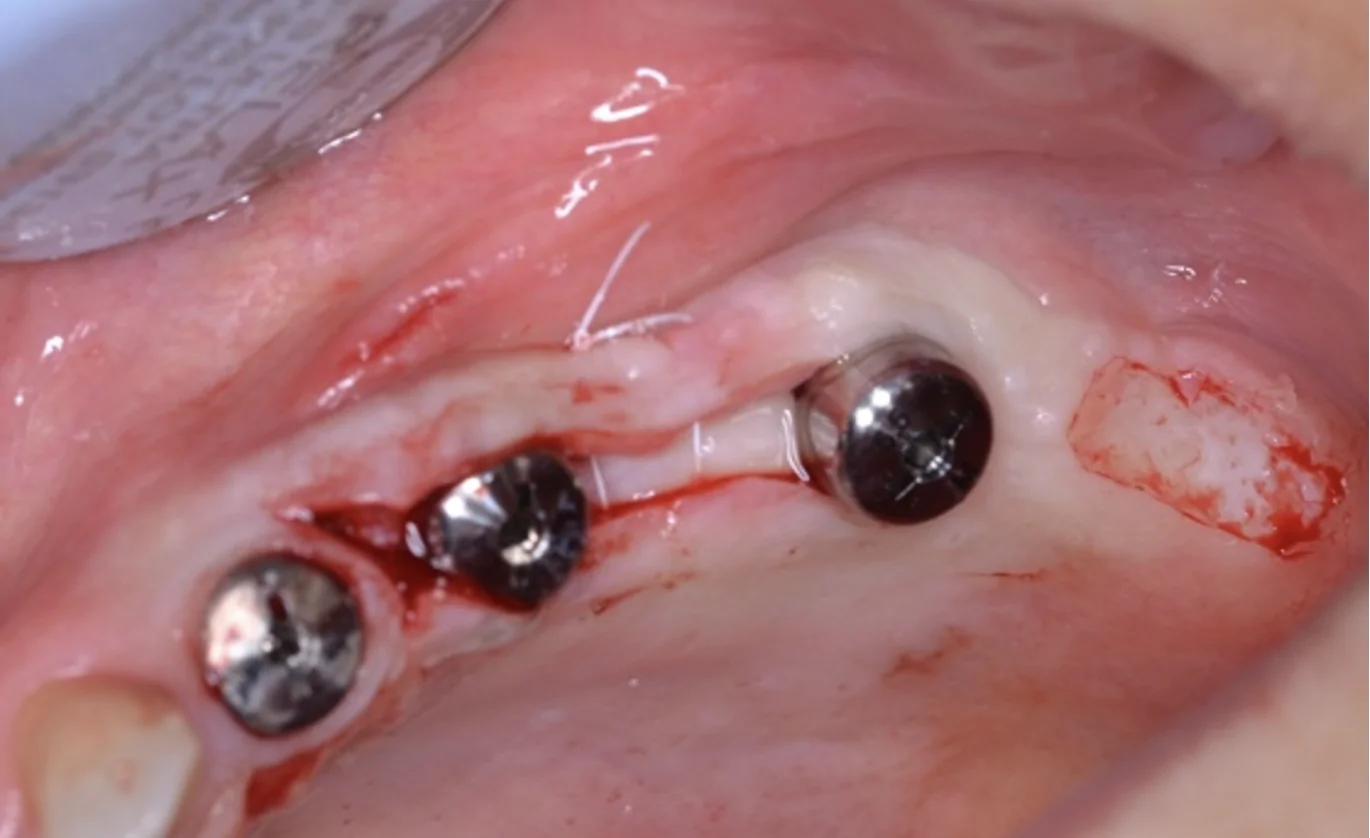
Intraoperative view at the second stage (implant uncovering).

**Fig 3b:**
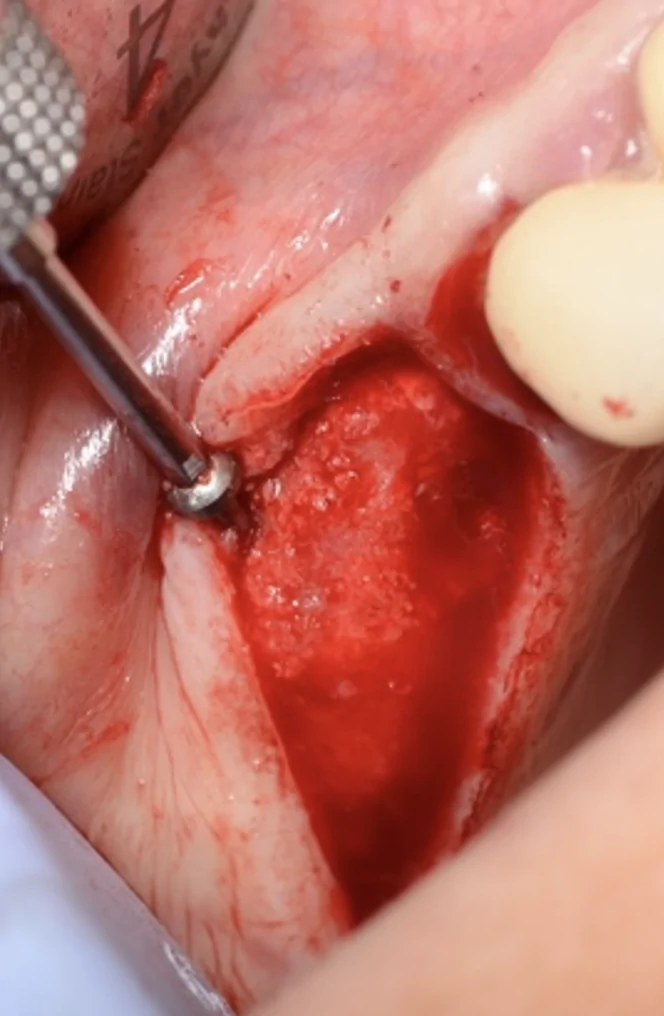
Intraoperative view at the second stage (implant uncovering).

**Fig 4b:**
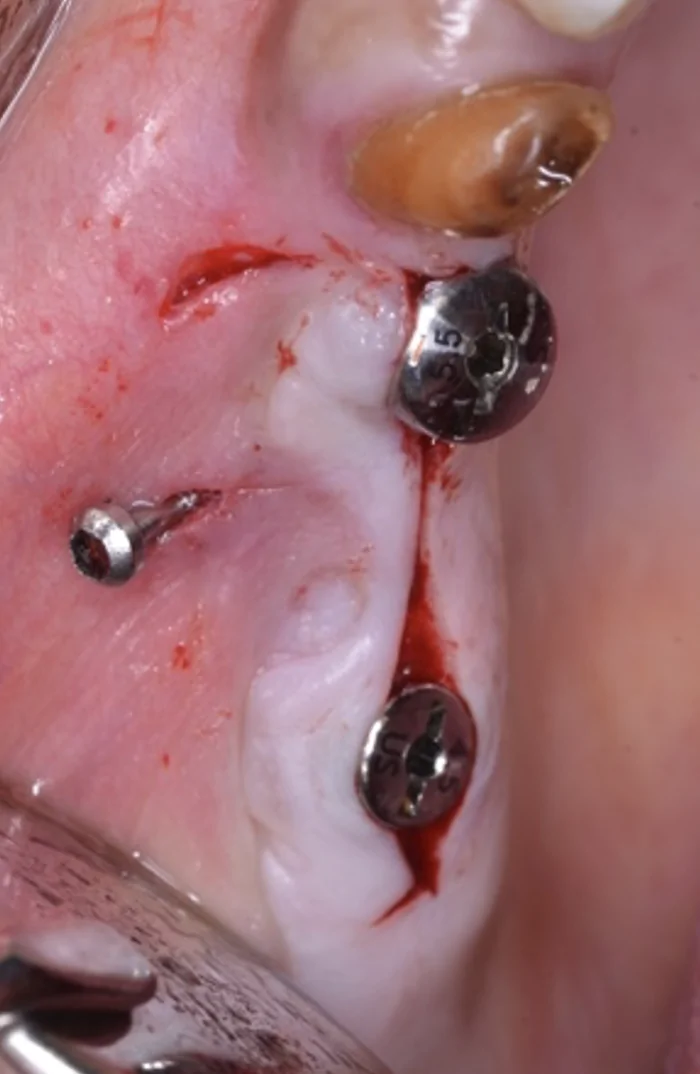
Intraoperative view at the second stage (implant uncovering).

**Fig 5b:**
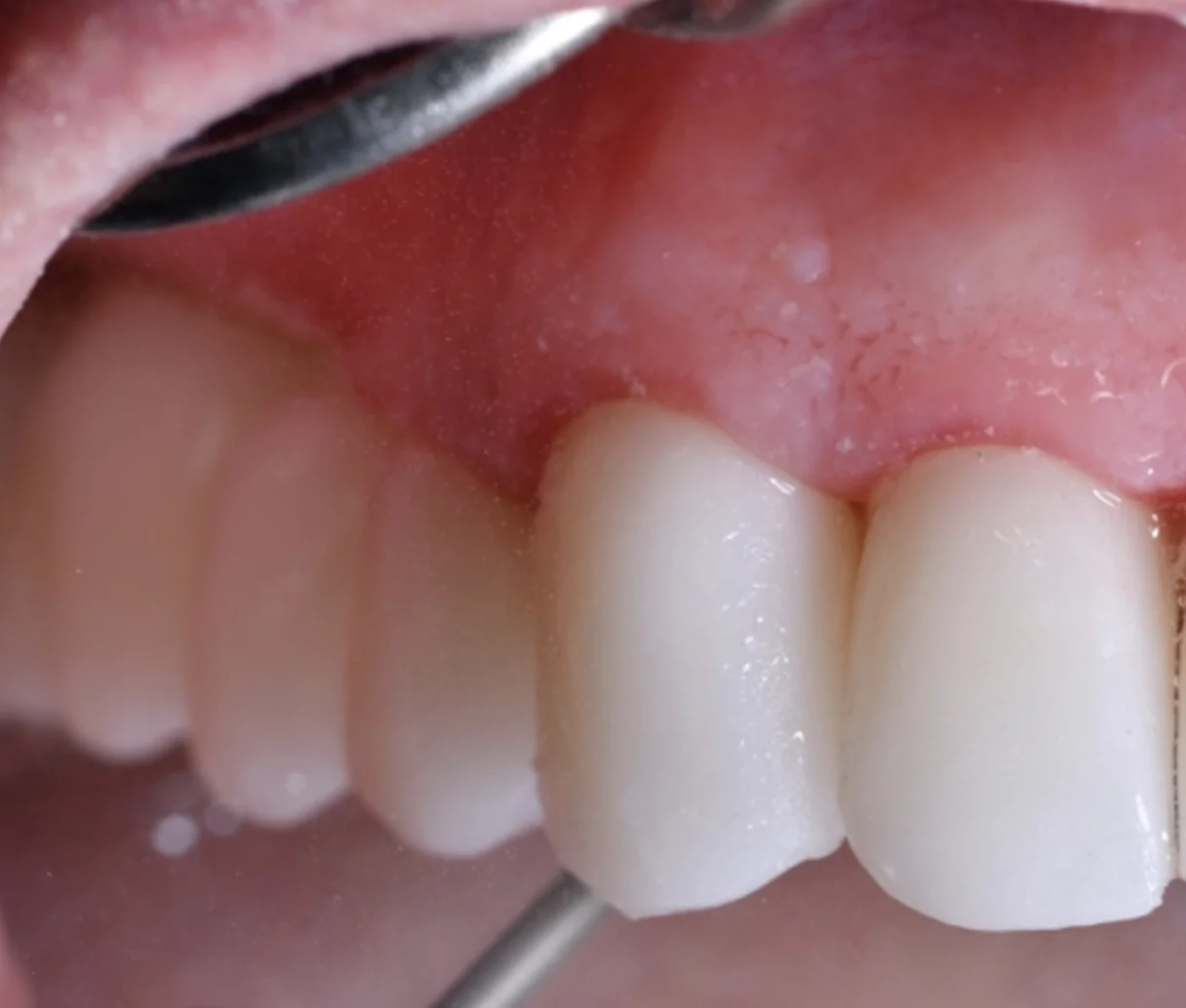
Clinical situation at follow-up (18 months).

**Fig 6b:**
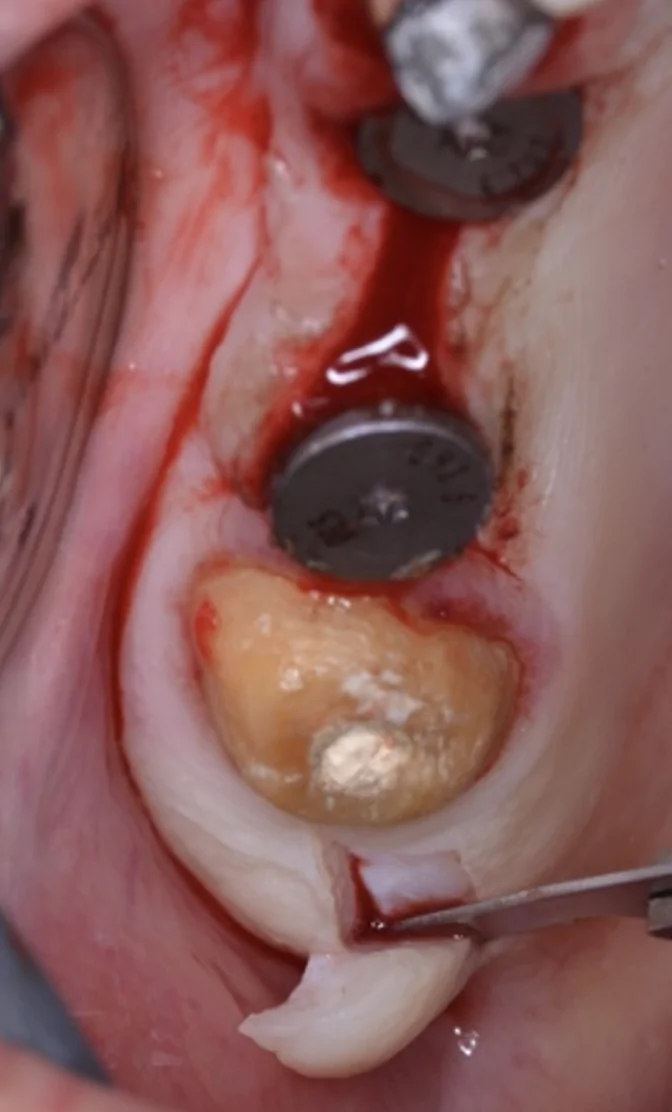
Intraoperative view at the second stage (implant uncovering).

**Fig 7b:**
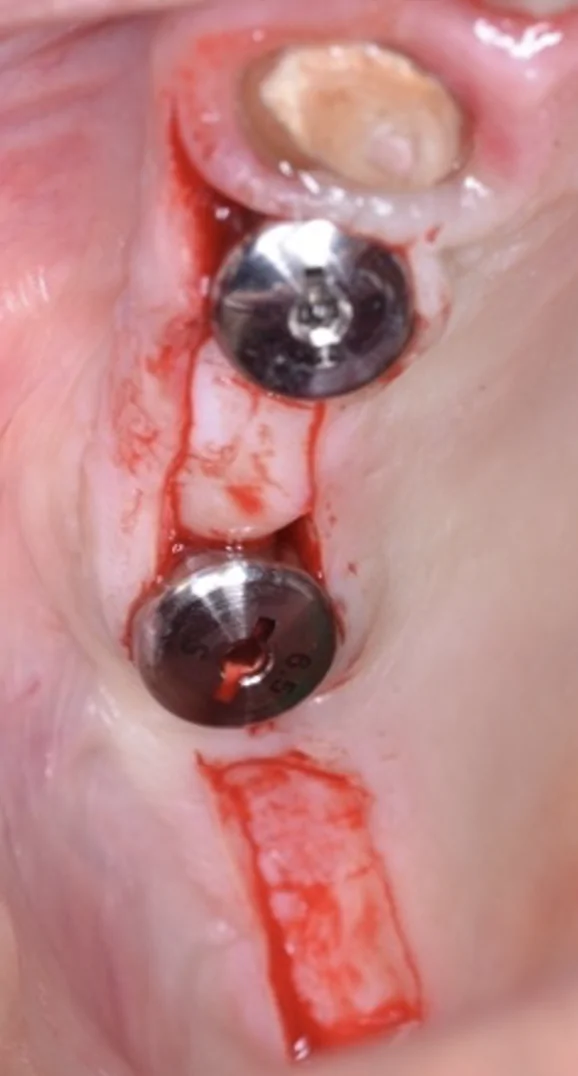
Intraoperative view at the second stage (implant exposure).

**Fig 8b:**
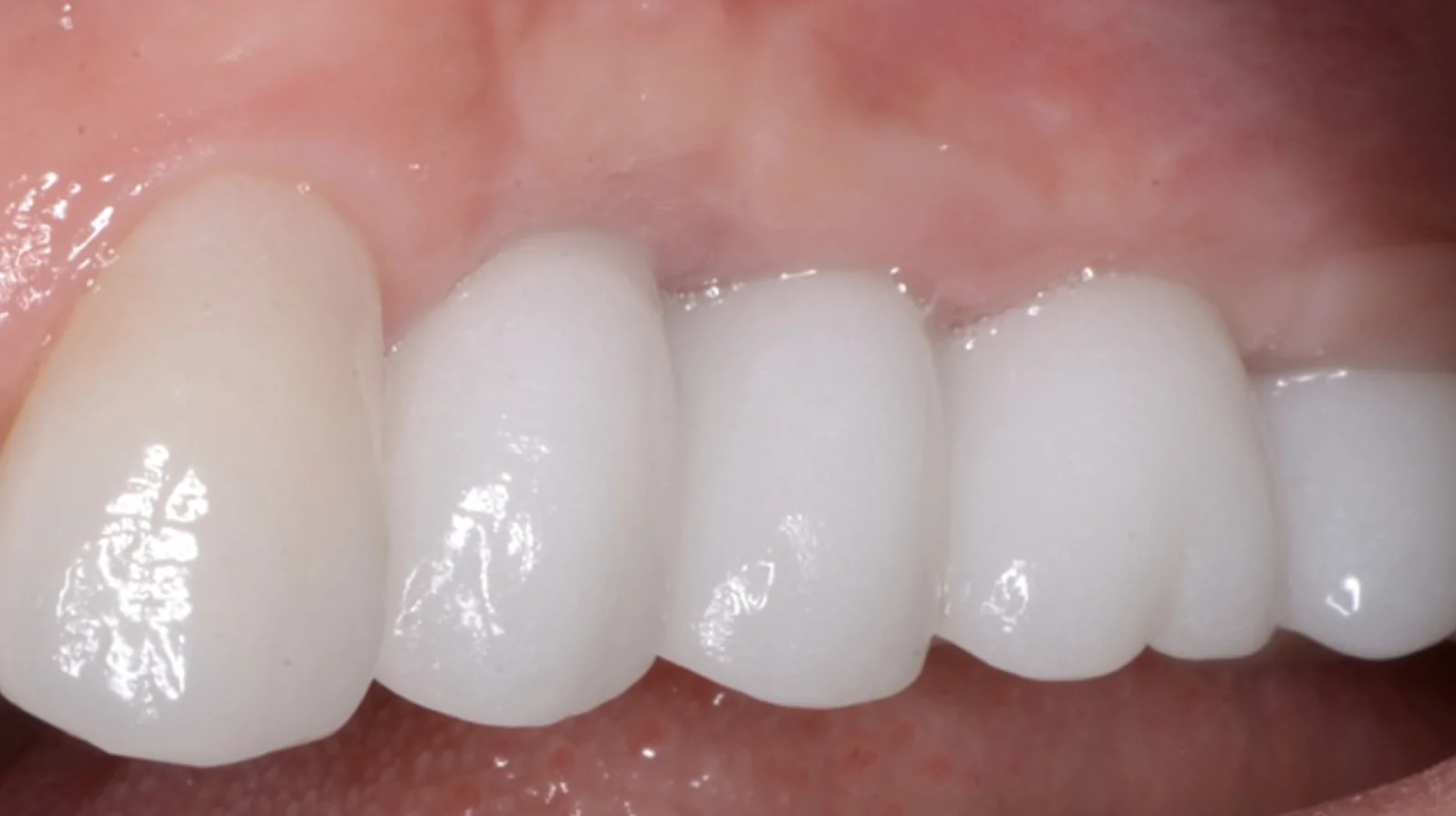
Clinical situation at follow-up (36 months).

All surgeries were performed as follows: a crestal incision with papilla preservation was performed and a full-thickness flap delimited by two vertical releasing incisions was raised. Thereafter, an external sinus lift elevation was conducted by preparing a bony window at the sinus level using piezo instruments (Piezotome CUBE and Piezo Kit, Acteon; Mérignac, France) followed by elevation of the thin lateral window (Figs 1s to 8a).^42^ The lateral bony window was then harvested using piezoelectric instruments and stored in sterile saline solution until used for the lateral ridge augmentation step. After elevation of the Schneiderian membrane (Figs 1a to 8a), the sinus was augmented using bovine (3 patients, BioOss [Geistlich Biomaterials; Wolhusen, Switzerland] granulation 0.25-1mm, 1-2 mm) or porcine (5 patients, THE Graft [Purgo Biologics; Seongnam-si, Gyeonggi-do, Korea], granulation 0.25-1mm, 1-2mm) natural bone mineral graft (Figs 1a to 8a) (Table 1). Based on the previous prosthetic analysis and 3D-planning, 2-5 implants were inserted in the required positions (Table 1, Figs 1a to 8 a). The sinus area was then covered with a collagen membrane (3 patients received BioGide 30/40 mmM 5 patients received THE Cover Flex 25/30 mm, Purgo, Table 1).

**Fig 2c:**
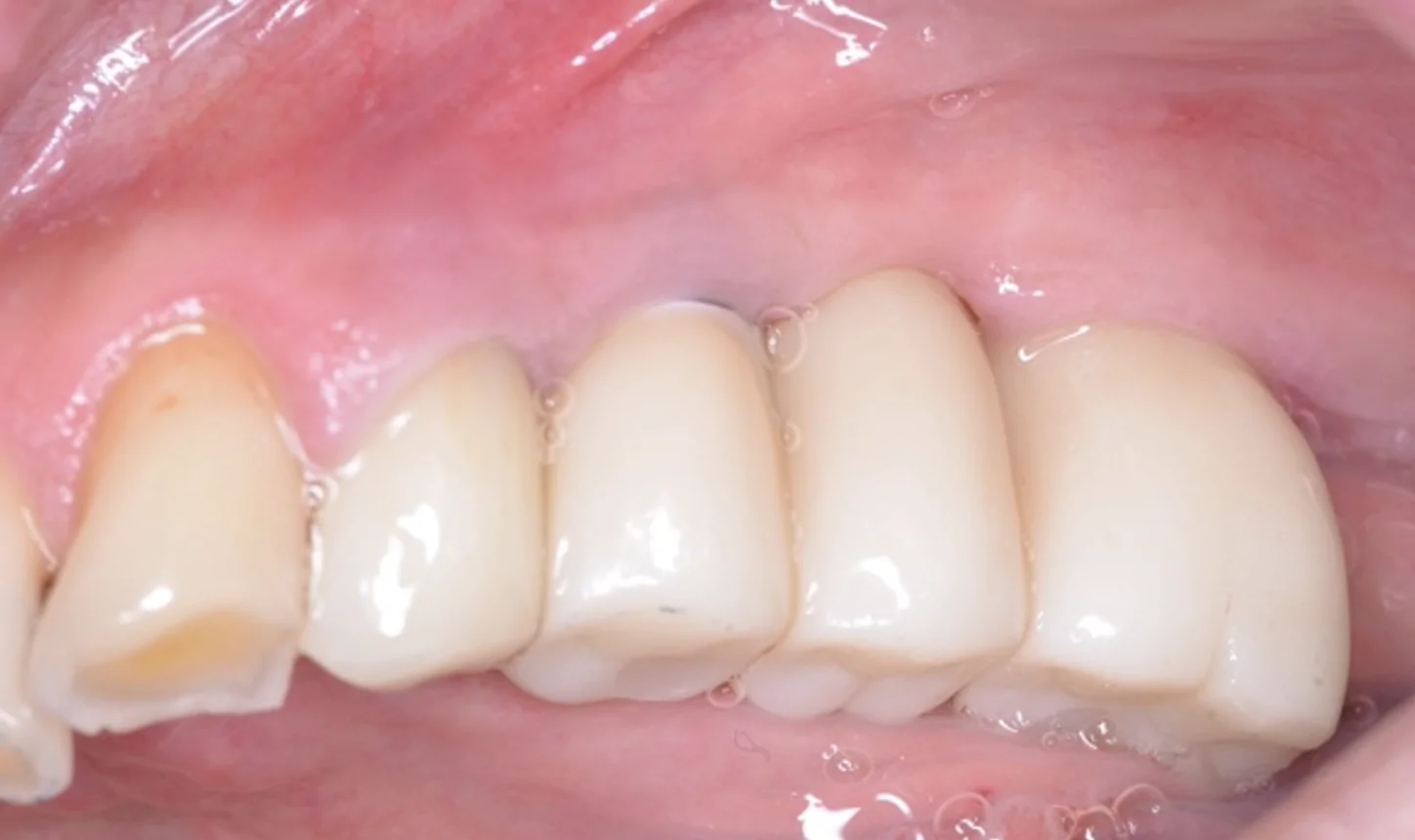
Clinical situation at follow-up (18 months).

**Fig 3c:**
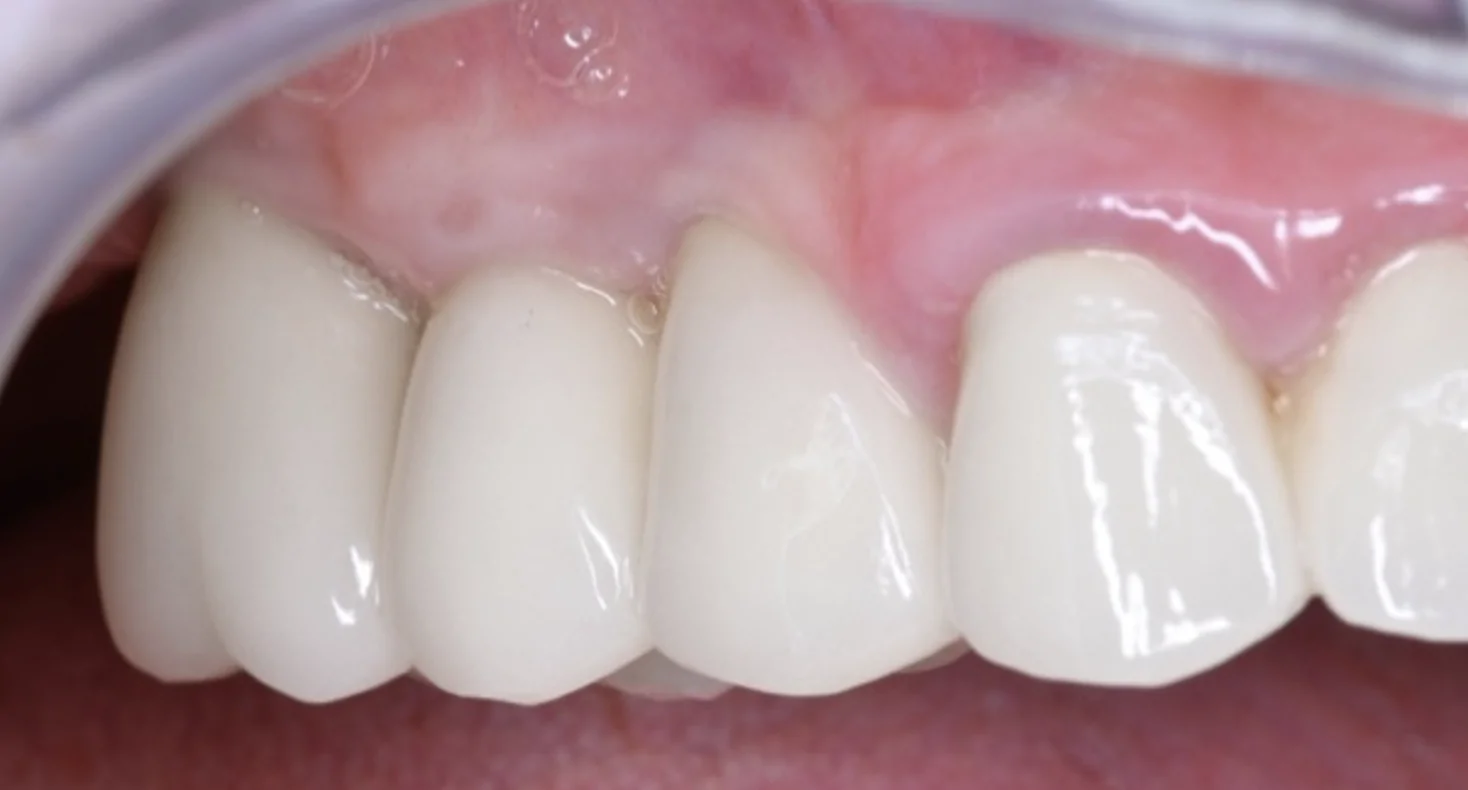
Clinical situation at follow-up (18 months).

**Fig 4c:**
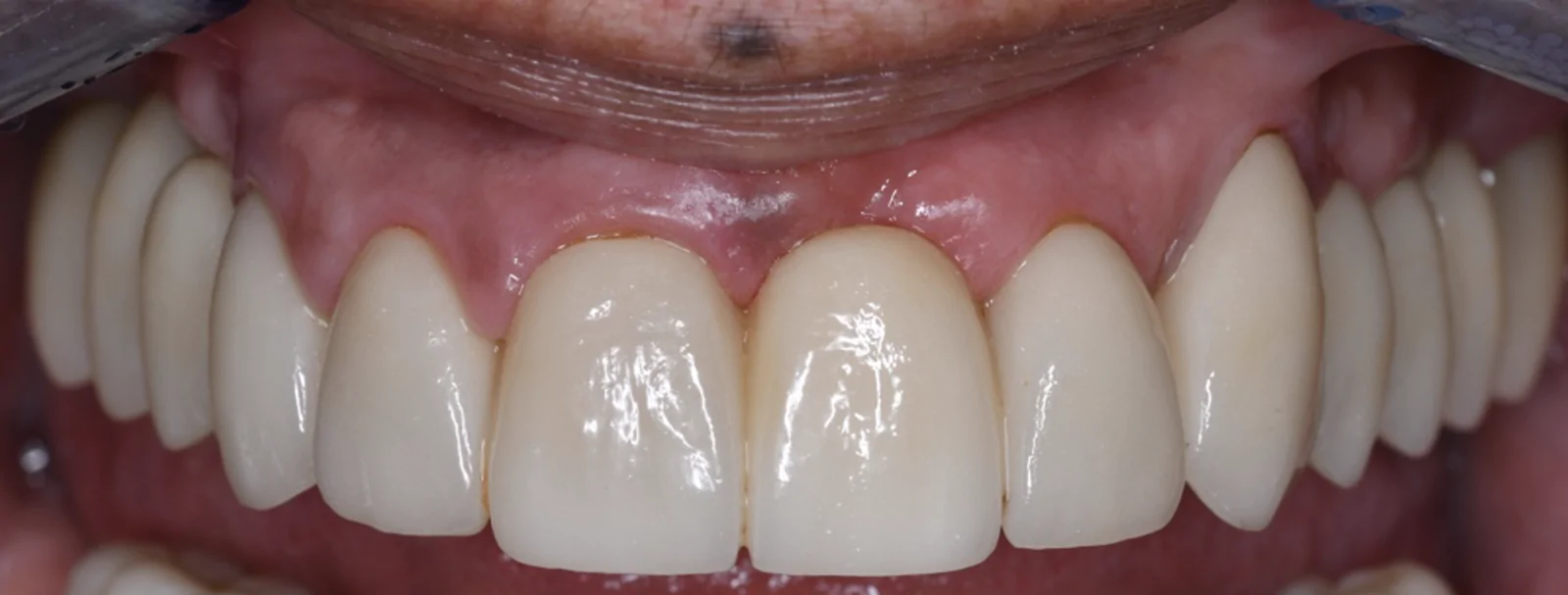
Clinical situation at follow-up (18 months).

**Fig 6c:**
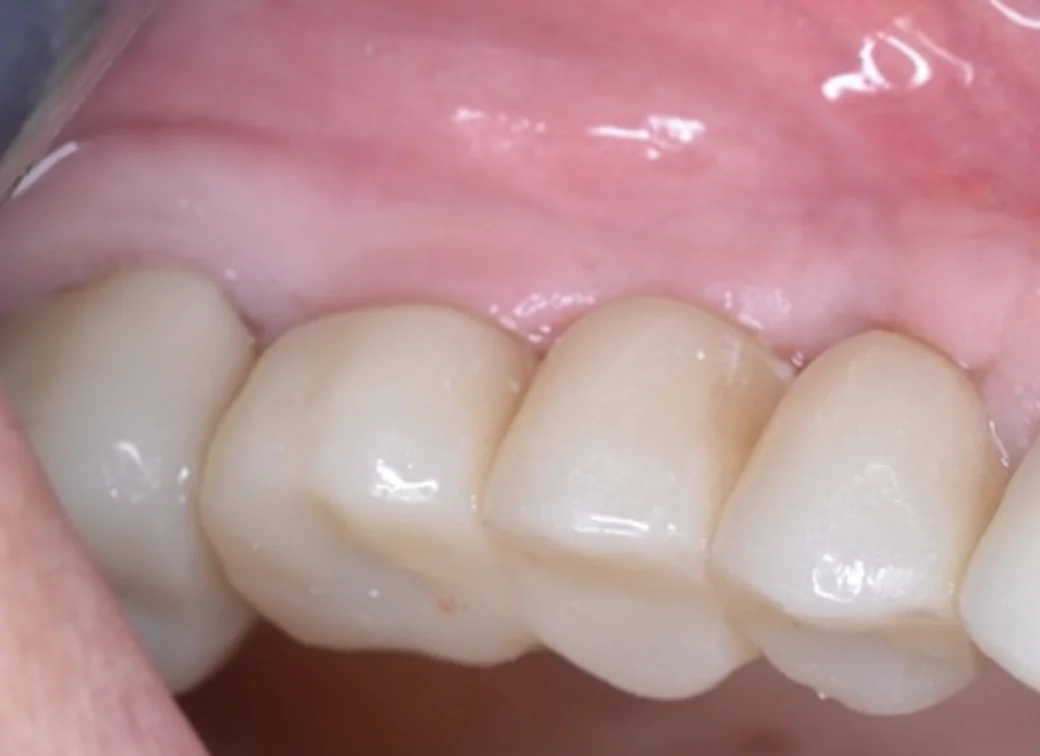
Clinical situation at follow-up (18 months).

**Fig 7c:**
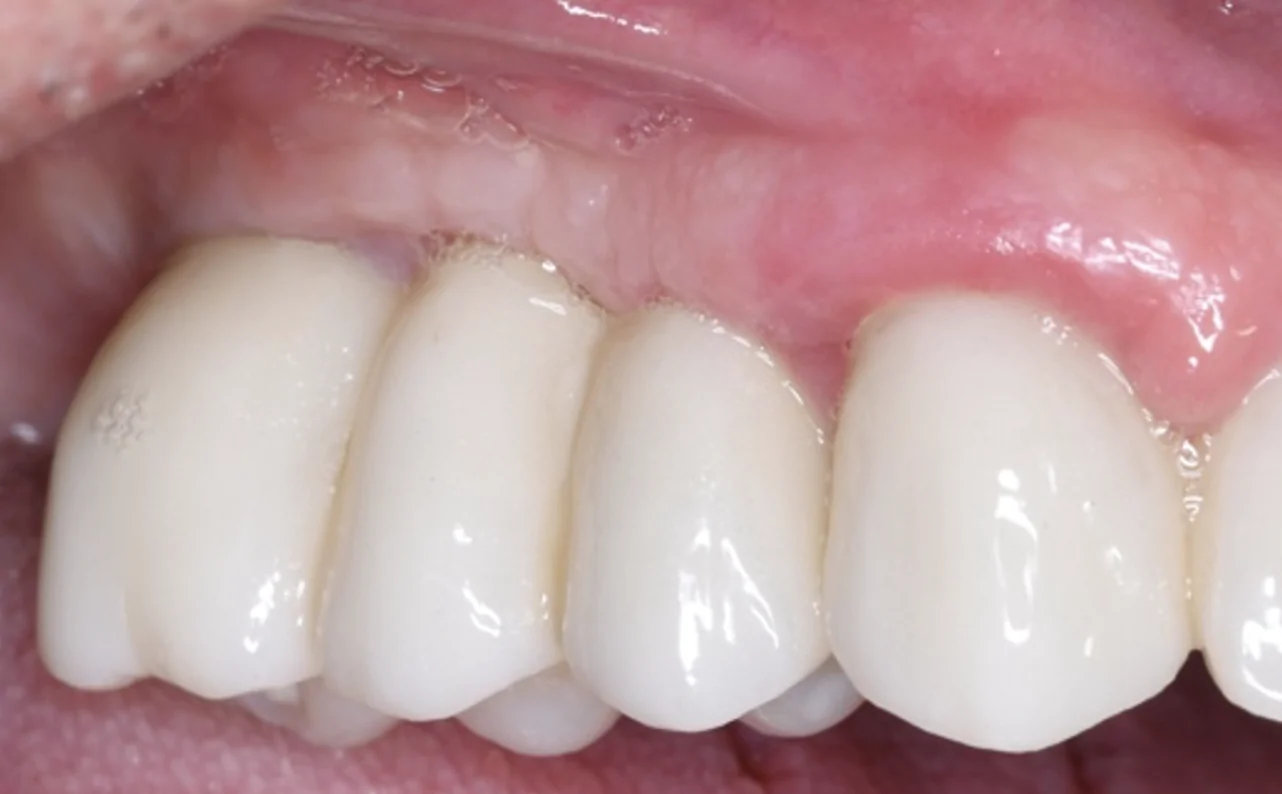
Clinical situation at follow-up (12 months)

**Table 1:** Demographic data and used materials for the eight patients

Variables	Values	Inserted implants ∅/ length (mm) Manufacturer	Regenerative materials for augmentation
Gender: Female/Male (n)	7/1	-	-
Age in years (mean±SD)	57.37 ± 7.44	-	-
Smokers (n)	2	-	-
Alveolar ridge resorption (after Allen 1985)^4^ (n patients) Degree A	0	-	-
Degree B	0	-	-
Degree C	8	-	-
FMPS (%)	<30%	-	-
**N/ area inserted implant**			
Patient 1	n=3/ 23, 24, 26	3.9/ 13, 3.3/ 11.5 MIS V3; Bar-Lev, Israel	Porcine natural bone mineral + porcine collagen membrane (0.25-1 mm, 1-2 mm, THE Graft, Purgo; Seongnam-si, Gyeonggi-do, Korea)
Patient 2	n=3/ 23, 24, 26	3.6/ 12, 3.6/ 12, 3.6/ 12 Dentium Superline; Seoul, Korea	Porcine natural bone mineral + porcine collagen membrane (0.25-1 mm, 1-2 mm, THE Graft, Purgo)
Patient 3	n=4/ 16, 14, 24, 26	3.75/ 11.5, 3.75/ 11.5, 3.75/ 11.5, 3.75/ 11.5 MIS Seven; Bar-Lev, Israel	Porcine natural bone mineral + porcine collagen membrane (0.25-1 mm, 1-2 mm, THE Graft)
Patient 4	n=2/ 14, 16	3.6/ 12, 3.6/ 12 Dentium Superline	Porcine natural bone mineral + porcine collagen membrane (0.25-1mm, 1-2mm, THE Graft)
Patient 5	n=2/ 15, 17	3.75/ 14, 4/14 Straumann BLX; Basel, Switzerland	Bovine natural bone mineral + bovine collagen membrane (0.25-1 mm BioOss, BioGide 30/40 mm, Geistlich Biomaterials; Wolhusen, Switzerland)
Patient 6	n=2/ 14, 16	3.5/12, 3.75/12 Straumann BLX	Bovine natural bone mineral + bovine collagen membrane (0.25-1 mm BioOss, BioGide 30/40 mm, Geistlich Biomaterials)
Patient 7	n=5/ 14, 16, 23, 25, 27	3.6/16, 3.6/16, 4/11, 4/13, 4.5/13 Dentium Bright; Seoul, Korea	Porcine natural bone mineral + porcine collagen membrane (0.25-1 mm, 1-2 mm, THE Graft, Purgo)
Patient 8	n=3, 23, 25, 27	3.75/11.5, 3.75/11.5, 4.2/11.5 Mis Seven; Bar-Lev, Israel	Bovine natural bone mineral + porcine collagen membrane (0.25-1 mm BioOss, BioGide 30/40mm, Geistlich Biomaterials)
**Total**	**24 implants**		
Follow-up time (months)			
Patient 1	18m	-	-
Patient 2	18m	-	-
Patient 3	18m	-	-
Patient 4	18m	-	-
Patient 5	18m	-	-
Patient 6	18m	-	-
Patient 7	12m	-	-
Patient 8	12m	-	-
m: months, n: number; y= years; FMPS: full-mouth plaque scores; ∅: diameter.			

Thereafter, the previously harvested autologous bone plate from the lateral sinus window was fixed to the alveolar bone with a 8- to 10-mm-long fixation screw (Meisinger; Neuss, Germany). The space between the bony plate and the alveolar bone was filled up with natural bovine/porcine bone mineral grafting material (BioOss in 3 patients, granulation 0.25-1 mm, 1-2 mm; THE Graft in 5 patients, granulation 0.25-1 mm, 1-2 mm) and the entire augmented area was covered with a bioresorbable collagen porcine membrane (BioGide in 3 patients, 30/40 mm; THE Cover Flex in 5 patients,-25/30 mm) (Table 1, Figs 1a to 8 a). Tension-free flap adaptation was assured by periosteal incision and sectioning of the inserting muscle fibers at the inner aspect of the flap. The collagen membrane was fixed with mattress sutures (PTFE suture material, Coreflon; Poznan, Poland) at the palatal and vestibular flaps (Figs 1a to 8a), and the surgical site was then sutured tension-free so as to assure primary wound healing (Figs 1a to 8a).

Post-operatively, all patients rinsed with a chlorhexidine digluconate solution (0.12%) twice daily for two weeks and abstained from brushing in the operated area. Additionally, antibiotic prophylaxis with amoxicillin (875 mg) and clavulanic acid (125 mg) (Augmentin, GlaxoSmithKline; London, UK) and anti-inflammatory therapy (Ibuprofen 400 mg/8h) were prescribed for seven days. All sutures were removed two weeks post-surgically.

Selection of the implant and augmentation material brands was based on the financial means of the patients.

Follow-up visits were performed at 6, 12, and 18 months for six patients (Figs 1c to 8c), while for one patient only at 6 and 12 months, and for another at 6 and 36 months. Each follow-up consisted of clinical examination of the implant area as well as assessment of oral hygiene, peri-implant bleeding, and probing depth. Final prosthetic reconstructions were performed 6-12 months postoperatively by another dentist.

### CBCT Measurements

CBCT scans were taken pre- and postoperatively with the same CBCT unit in a standardized manner (standardized parameters, fixed patient position). To ensure that bone-implant regions on CBCT slices were consistently identified across the different timepoints, reference landmarks on neighboring teeth and standardized plane orientation were used.

Horizonal alveolar bone width was measured in the vestibulo-oral direction pre- and post-operatively, as well as at 12 to 36 months after surgery at the implant shoulder level on CBCT images using OnDemand3D software (CyberMed) by one single examiner (Figs 1d to 8c) (Table 2). Intra-examiner reliability had already been determined on previous scans.

**Table 2:** Radiographic analysis before, post-operatively and at the follow-up (12-36 months post-operatively) performed on CBCT sections

Alveolar bone width	Pre-operative Mean±SD (mm)	Post-operative Mean±SD (mm)	Follow-up 12-36 months Mean±SD (mm)	Lateral bone gain at 12-36 months Mean±SD (mm)
Patient 1	5.43±1.74	10.16±3.58	9.46±2.51	4.03±3.72
Patient 2	5.7±1.21	8.7±3.26	7.6±2.68	1.9±1.95
Patient 3	4.95±0.95	8.57±1.35	8.86±2.85	3.91±2.67
Patient 4	4.65±1.20	7.2±0.42	5.5±0.98	0.85±0.21
Patient 5	9.95±2.19	10.62±2.19	10.5±1.27	0.65±0.77
Patient 6	5.85±2.33	9.52±1.52	7.77±0.95	1.9±1-37
Patient 7	4.84±1.84	10.74±2.09	8.16±2.21	4.52±0.78
Patient 8	6.6±2.4	8.8±1.34	9.16±1.36	2.56±1.17
Total	5.99±1.71	9.28±1.20	8.37±1.50	2.55±1.47


## RESULTS

A total of 24 implants in 8 patients were inserted simultaneously with the SPAM technique and sinus-lifting procedure. The patients were followed-up for 12 to 36 months based on the patients’ ability to attend the follow-up (see Table 1 for details).

Post-operative healing was uneventful in all patients, without any complications at the grafted sites. Symptoms like tenderness and light to moderate swelling were present up to 3-5 days in all patients.

Bone level changes ranged on average from 5.99 ± 1.71 mm preoperatively to 9.28±1.20 mm post-operatively, and to 8.37 ± 1.50 mm at the 12- to 36-month follow-ups (Figs 1d to 8c). Some resorption in the augmented area (9.8%, 0.91 ± 1.06 mm) due to bone remodeling could be observed between the immediate postoperative timepoint and the follow-up (from 9.28 ± 1.20 mm to 8.37 ± 1.50 mm). However, a statistically significant gain in alveolar bone width of 2.55 ± 1.47 mm was present at 12 to 36 months (mean 19.5 ± 6.98) postoperatively (Table 2). In all patients at each follow-up, the clinical examination revealed a healthy peri-implant status with PPD ≤5mm, and no profuse peri-implant bleeding on probing. No iimplants were lost (100% implant survival) and no biological (i.e., peri-implant mucositis, peri-implantitis, vertical bone loss above the expected bone remodeling ≥1mm) or prosthetic complications (i.e., ceramic fracture, abutment fracture/loosening, decementation) occurred within the evaluated follow-up timeframe.

## DISCUSSION

This case series presents a new surgical approach for concomitant implant insertion with external sinus lifting and lateral bone augmentation in the edentulous posterior maxillary area with severe alveolar bone resorption using the external sinus bony wall and xenogeneic natural bone mineral. In all eight patients, healing occurred without complications, and a statistically significant mean gain in alveolar bone width (2.55 ± 1.47 mm) was observed on CBCT at a follow-up mean of 19.5 ± 6.98 months.

Comparable amounts of gain in alveolar bone width (up to 3.7 ± 1.8 mm) and higher values of bone resorptions of the augmented sites up to 2. 3± 1mm (as opposed to 0.91 ± 1.06 mm, 9.8% in the current case series) were reported in various clinical studies that used resorbable/non-resorbable membranes and autologous grafts.^6,21,22,30,33^ High-volume loss after bone augmentation for autologous grafts is a common finding in the edentulous atrophic maxilla. Furthermore, another systematic review reported volume losses of 5% to 50% for sinus inlay grafts and 5% to 47% for lateral bone augmentation procedures using autogenous iliac bone grafts, particulated autogenous bone grafts, or monocorticocancellous bone blocks mixed or not with platelet-rich-plasma, or xenografts.^12^

Autogenous bone blocks have been recommended by various research groups for the reconstruction of significant horizontal alveolar bone defects,^2,11,26,31^ with or without allograft biomaterials, depending on the defect size and autogenous bone availability.^28^ The combination of the autogenous bone provided by the bony window with a collagen barrier membrane ensures the creation of a space where only cells from the adjacent bone will migrate into the bone defects, while avoiding the proliferation of soft tissue cells from the overlying mucosa, mechanical disruption, and salivary contamination.^27^ Depending on the required graft structure and needed bone volume, various research groups recommend donor sites such as ramus grafts, block bone grafts from the posterior iliac crest, or cancellous bone grafts harvested from the anterior iliac crest.^34^ All these techniques involve a second surgical site with a considerable increase in the patients’ morbidity. As opposed to these techniques, the greatest advantage of the SPAM technique is the avoidance of a secondary surgical site. Additionally, complications seem to be more frequent when bone grafts are provided by iliac crest donor sites.^15,29^ Further advantages of SPAM are related to the reduced morbidity, avoiding a second surgical site, and the possibility of simultaneous horizontal and vertical augmentation. Moreover, the reduced amount of additionally harvested autogenous bone needed for the augmentation (since the sinus cortical window plate is used as a shield) also contributes to a lower morbidity. From the surgical point of view, due to the curved anatomy of the harvested bony sinus window plate, easier fixation of the block may be possible.

Various results have been reported for sinus lifting combined with alveolar bone augmentation techniques using autogenous bone with/without xenograft/allograft biomaterials. In cases with posterior atrophic maxillae with residual heights of 3-6 mm, more complications were reported when sinus lift augmentation was performed with autogenous bone grafts as opposed to augmentation based on autologous/xenograft combinations for the reconstruction of implant sites by the lateral approach technique.^9^ Sbordone et al^37^ reported 1.5 years post-operatively similar volumetric changes for sinus augmentation with autogenous bone blocks or with freeze-dried allogeneic bone (FDBA) harvested from the iliac crest. Xavier et al^46^ compared volumetric changes after bilateral sinus grafting with autogenous or allogeneic fresh-frozen bone particles. Based on computed tomography scan measurements, comparable volume changes were reported after 12 months (31.35% for autogenous grafts; 35.36% for allogeneic grafts).^46^

Volumetric contractions after sinus floor augmentation methods using deproteinized bovine bone mineral were reported by other authors as well. Zhang et al^48^ observed after 6 months a height reduction of 17.8% and a volumetric contraction of 22.7%. Those authors also indicated that the type of edentulism (single vs multiple lost teeth) was statistically significantly associated with the contraction volume. On the other hand, height reduction was at each follow-up statistically significantly influenced by the width of the sinus floor and the angle between the medial and lateral sinus wall.^48^ Another review evaluating the efficacy of various grafting materials in alveolar ridge augmentation observed a higher horizontal gain (approx. 1 mm) with the use of autologous block grafts.^43^ Moreover, when these were extraorally extracted, a statistically significantly higher vertical gain was obtained compared to particulate material.^43^ Another retrospective study supporting the use of autogenous bone blocks reported a higher vertical gain for autogenous bone blocks compared to allogenous bone blocks or particulate material.^35^ While autogenous block grafts undergo rapid remodeling and significant volume contraction within the first year, alternative biomaterials exhibit slower degradation, requiring a longer period to integrate biologically but offer superior early dimensional stability.^35^ Other adjunctive materials that may improve dimensional stability include the use of a membrane. A recent meta-analysis indicated that postoperative dimensional changes of augmented sites using autogenous bone blocks may be diminished when barrier membranes are used.^21^ These differences were, however, statistically non-significant.

Nevertheless, the current evidence indicates no clear superiority of one GBR technique over another, and the decision for the treatment of choice (e.g., technique, biomaterials) should be based on the risks of complications as well as patient- and defect-related factors.^8^

Risks and mechanical considerations for this technique imply the risk of plate fracture in cases where the plate is very thin and the screw is to tightly drawn; the stability of the fixed plate as well as that of the implant may also be problematic in cases where primary stability is impossible or where sufficient fixture of the plate is not achievable due to fracture risk of the bony plate. Moreover, the expected bone loss due to remodeling processes has to be accounted for when augmenting these areas. Consequently, careful case selection is critical for the success of simultaneous implantation with SPAM.

This study has some limitations: relatively few patients (8), non-consecutive sampling procedure, material heterogeneity. and variable follow-up. Therefore, the current results must be interpreted with care. Further well-designed studies evaluating the technique described here in larger study cohorts, as well as randomized comparisons with other GBR techniques used adjunctively with sinus lifting, are required to validate and support the implementation of the SPAM technique during the pre-implant stage of implant-prosthetic therapy.

## CONCLUSION

The use of autologous bone harvested from the lateral bony sinus window prepared during the open-window sinus lifting technique may represent an valuable option for the reconstruction of severely resorbed posterior maxillary alveolar areas in patients requiring implant-prosthetic therapy. The absence of biological complications, together with the gain in alveolar bone width and its maintenance for up to 19.5 months, supports the potential of the SPAM technique for the reconstruction of future implant sites in posterior maxillary regions.

**Fig 1:** Clinical surgical procedure and radiographical analysis of Patient 1.

**Fig 1a1 to 1a7:** Surgical procedure(left-right): preparation and elevation of the lateral sinus bony wall, fixture of the harvested bony wall to the alveolar ridge, lateral ridge augmentation with particulate xenogeneic bone graft material, suturing of the collagen membrane, suturing of the flap.

**Fig 1d1:** Radiographical analysis: pre-operatively.

**Fig 1d2:** Radiographic analysis: post-operatively.

**Fig 1d3:** Radiographic analysis: at follow-up (18 months).

**Fig 2:** Clinical surgical procedure and radiographical analysis of Patient 2.

**Fig 2a1 to 2a5:** Surgical procedure (left-right): incision, preparation and elevation of the lateral sinus bony wall, socket preparation for implant insertion, lateral ridge augmentation with particulate xenogeneic bone graft material, sutures.

**Fig 2d1:** Radiographic analysis: pre-operatively.

**Fig 2d2:** Radiographic analysis: at follow-up (18 months).

**Fig 3d:** Radiographic analysis (pre-, post-operative and at follow-up).

**Fig 3d1:** Radiographic analysis: pre-operatively.

**Fig 3d2:** Radiographic analysis: post-operatively.

**Fig 3d3:** Radiographic analysis: at follow-up (18 months).

**Fig 3:** Clinical surgical procedure and radiographical analysis of Patient 3.

**Fig 3a1 to 3a7:** Surgical procedure (left-right): preparation and elevation of the lateral sinus bony wall, fixture of the harvested bony wall to the alveolar ridge, adaptation of the lateral bony wall, socket preparation for implant insertion, fixture of the harvested bony wall to the alveolar ridge, lateral ridge augmentation with particulate xenogeneic bone graft material.

**Fig 4d:** Radiographic analysis (pre-, post-operative and at follow-up).

**Fig 4d1:** Radiographic analysis: preoperatively.

**Fig 4d2:** Radiographic analysis: post-operatively.

**Fig 4d3:** Radiographic analysis: at follow-up (18 months).

**Fig 4:** Clinical surgical procedure and radiographical analysis of Patient 4.

**Fig 4a1 to 4a6:** Surgical procedure(left-right): preparation of the lateral sinus bony wall, socket preparation for implant insertion, fixture of the harvested bony wall to the alveolar ridge, lateral ridge augmentation with particulate xenogeneic bone graft material, suturing of the flap.

**Fig 5:** Clinical surgical procedure and radiographical analysis of Patient 5.

**Fig 5a1 to 5a5:** Surgical procedure(left-right): preparation of the lateral sinus bony wall, socket preparation for implant insertion, fixture of the harvested bony wall to the alveolar ridge and lateral ridge augmentation with particulate xenogeneic bone graft material, suturing of the flap.

**Fig 5c:** Radiographic analysis (pre-, post-operative and at follow-up).

**Fig 5c1:** Radiographic analysis: preoperatively.

**Fig 5c2:** Radiographic analysis: post-operatively.

**Fig 5c3:** Radiographic analysis: at follow-up (18 months).

**Fig 6:** Clinical surgical procedure and radiographical analysis of Patient 6.

**Fig 6a1 to 6a7:** Surgical procedure(left-right): preparation of the lateral sinus bony wall, socket preparation for implant insertion, augmentation of the sinus area, fixture of the harvested bony wall to the alveolar ridge, lateral ridge augmentation with particulate xenogeneic bone graft material, suturing of the collagen membrane, suturing of the flap.

**Fig 6d:** Radiographic analysis (pre-, post-operative and at follow-up).

**Fig 6d1:** Radiographical analysis: preoperatively.

**Fig 6d2:** Radiographical analysis: post-operatively.

**Fig 6d3:** Radiographic analysis: at follow-up (18 months).

**Fig 7:** Clinical surgical procedure and radiographical analysis of Patient 7.

**Fig 7a1 to 7a6:** Surgical procedure(left-right): preparation and elevation of the lateral sinus bony wall, fixture of the harvested bony wall to the alveolar ridge, lateral ridge augmentation with particulate xenogeneic bone graft material, suturing of the collagen membrane, suturing of the flap.

**Fig 7d:** Radiographic analysis (pre-, post-operative and at follow-up).

**Fig 7d1:** Radiographic analysis: preoperatively.

**Fig 7d2:** Radiographic analysis: post-operatively.

**Fig 7d3:** Radiographic analysis; at follow-up (18 months).

**Fig 8:** Clinical surgical procedure and radiographical analysis of Patient 8.

**Fig 8a1 to 8a8:** Preparation and elevation of the lateral sinus bony wall, socket preparation for implant insertion, sinus augmentation with xenogeneic particulate bone material, lateral ridge augmentation with xenogeneic bone graft, suturing of the collagen membrane, suturing of the flap.

**Fig 8c:** Radiographic analysis (pre-, post-operative and at follow-up).

**Fig 8c1:** Radiographic analysis: preoperatively. post-operatively and at follow-up).

**Fig 8c2:** Radiographic analysis: post-operatively.

**Fig 8c3:** Radiographic analysis: at follow-up (36 months).




















































































































































